# Toothbrushing Ability in Older Adults Across Stages of Cognitive Impairment

**DOI:** 10.3390/geriatrics11040075

**Published:** 2026-06-25

**Authors:** Xi Chen, Jirakate Madiloggovit-Lower, Carissa Comnick, Daniel Tranel, Lisa Jacobson, Natalie Denburg

**Affiliations:** 1 College of Dentistry, The Ohio State University, Columbus, OH 43210, USA; 2 Faculty of Dentistry, Thammasat University, Pathum Thani 12120, Thailand; jirakate@tu.ac.th; 3 College of Public Health, University of Iowa, Iowa City, IA 52242, USA; caricomnick33@gmail.com; 4 Carver College of Medicine, University of Iowa, Iowa City, IA 52242, USA; daniel-tranel@uiowa.edu (D.T.); lisa-jacobson@uiowa.edu (L.J.); natalie-denburg@uiowa.edu (N.D.)

**Keywords:** oral self-care, toothbrushing, dental-related function, cognitive impairment, dementia, functional assessment, quality of oral self-care, older adults

## Abstract

**Background/Objectives:** Cognitive impairment can compromise toothbrushing and other oral self-care functions, increasing the risk of oral diseases and related complications. However, how toothbrushing ability declines across stages of cognitive impairment remains unclear. This study aimed to describe functional deficits in toothbrushing among older adults with different levels of cognitive function. **Method:** Sixty-five older adults (14 cognitively healthy and 51 with documented cognitive impairment) were classified into five cognitive levels based on Standardized Mini-Mental State Examination scores. Participants completed a toothbrushing task as they normally would at home. Performance was videotaped, coded, and evaluated across four domains (task initiation, completion, thoroughness, and quality) with total scores reflecting overall toothbrushing ability. Overall performance, functional deficits, and assistance needs were analyzed in relation to cognitive levels. **Results:** Participants averaged 76.5 years of age. Toothbrushing ability declined gradually with worsening cognitive impairment, followed by a sharp deterioration at the profound stage (e.g., SMMSE ≤ 5). Compared with cognitively healthy participants (*n* = 14), those with mild cognitive impairment (MCI, *n* = 20) or mild (*n* = 10), moderate (*n* = 10), or severe dementia (*n* = 11) lost an average of 3%, 8%, 12%, and 37% of overall toothbrushing ability, respectively. Brushing efficiency declined earlier and more rapidly, decreasing by 13% in MCI and up to 46% in severe dementia (*p* < 0.001). All participants with MCI or mild dementia completed the task independently, whereas 20% with moderate dementia and 80% with severe dementia required assistance to initiate or complete the task. **Conclusions:** Overall toothbrushing ability remains relatively preserved until the later stages of cognitive impairment, but brushing quality deteriorates much earlier and quicker. These findings highlight the importance of early caregiver–patient partnerships, functionally tailored oral self-care rehabilitation, and personalized caregiver training to support oral hygiene among older adults with cognitive impairment.

## 1. Introduction

Toothbrushing is an important activity of daily living (ADL) and is essential for maintaining oral health. Individuals typically begin learning and practicing toothbrushing in early childhood and gradually master their skills around the age of eight [1]. Proper toothbrushing helps remove food debris and dental plaque, a sticky bacterial film that plays a critical role in the development of caries and periodontal disease [2,3], from the teeth, the gum line and the surface of the tongue. When used with toothpaste, toothbrushing can also help reduce oral odors and keep breath smelling fresh. Therefore, toothbrushing plays a fundamental role in the prevention of dental caries, periodontal disease, and other oral diseases, supporting self-esteem and promoting social interaction [4,5,6,7,8].

**Figure 1:**
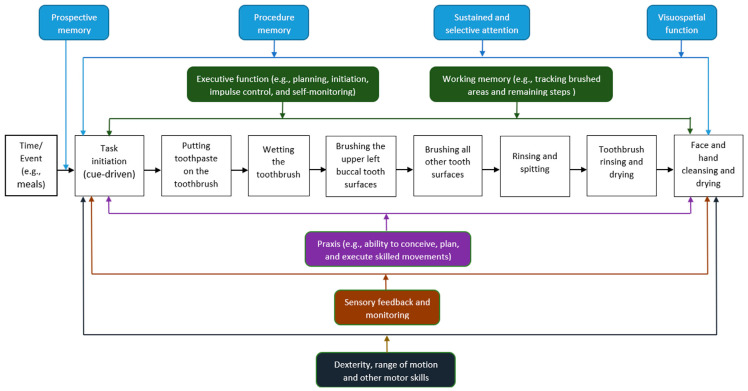
Conceptual framework of the cognitive mechanisms underlying toothbrushing in older adults with cognitive impairment. This model illustrates the sequential steps involved in toothbrushing and the underlying cognitive (blue and green), praxic (purple), sensory (brown), and physical (dark blue) domains that support performance. Time- or event-based cues (e.g., breakfast) trigger prospective memory, prompting task initiation. Executive functions, including planning, initiation, and sequencing, help structure the multi-step task. Attention, working memory, and visuospatial function enable monitoring and adaptive control. Praxic skills direct motor planning, while sensory and motor abilities support accurate execution. Disruption in any of these domains—common in dementia—can impair oral hygiene performance.

**Conceptual Framework:** Similar to many other ADLs, toothbrushing requires the integration of cognitive, motor, and sensory functions (Figure 1) [9,10]. For most people, toothbrushing is tied to specific daily events or routines (e.g., after breakfast and/or before bed), which act as cues to initiate the behavior. This process relies on event-based prospective memory, the ability to remember to perform an intended action when a specific event occurs (e.g., remembering to brush one’s teeth after eating) [11]. Meanwhile, several other cognitive domains are also involved [9,10,11]. Procedural memory, a type of long-term memory, enables retrieval of the learned motor routine necessary for toothbrushing. Sustained attention helps individuals remain focused throughout the task to ensure all teeth and surfaces are adequately brushed. Spatial and body awareness, as well as sensory-motor and perceptual-motor skills, support appropriate positioning and movement of the toothbrush, including adjusting angles to clean different surfaces effectively [12]. Furthermore, executive function is responsible for initiating, planning, and sequencing the toothbrushing task and ensuring that the task is performed in the planned order. Working memory, the ability to hold spatial and other information across multiple sensory channels in consciousness, helps individuals keep track of which areas have already been brushed and what steps remain. In addition, effective toothbrushing also requires fine motor skills, range of motion, eye–hand coordination, visual–motor and visual–perceptual abilities, as well as other physical and sensory functions, all of which contribute to the proper handling and maneuvering of the toothbrush [10,12]. Another key component is praxis, the neurological process by which cognition directs motor action. Praxis helps translate cognitive processes involved in toothbrushing into coordinated motor actions [13,14,15]. Specifically, it (1) conceives the idea, which is often triggered by a routine cue (e.g., eating breakfast); (2) plans the motor action, which depends on spatial and body awareness (e.g., the positions of the jaw, teeth and tongue and their relationships with the toothbrush); (3) executes the movements using coordinated action of the hand, jaw, tongue, and facial muscles; and (4) monitors and adapts the behavior based on sensory feedback (e.g., avoiding a sensitive area) [12,13,14,15]. Several components of executive functioning, including planning, initiation, impulse control, and self-monitoring, also are involved in implementing this multi-step process. For most people, it operates subconsciously as a result of repeated practice and reinforcement during early childhood [16,17].

Impairment in any of these domains can lead to altered toothbrushing patterns and decline in oral self-care function [9,10,11,12,13,14,18,19]. However, functional changes vary across levels of cognitive impairment. In mild cognitive impairment (MCI) or early-stage dementia, impairments in prospective memory, short-term memory and attention may emerge first, while procedural memory and motor routines are often preserved. Impaired prospective memory can weaken the link between a cueing event (e.g., eating breakfast) and the lifelong brushing habit. Consequently, the cueing event is less likely to trigger a toothbrushing activity as usual, leading to altered oral self-care patterns [11]. This, together with short-term memory loss and other neuropsychiatric symptoms (e.g., apathy or depression), can further disrupt oral hygiene routines [20,21]. As a result, individuals with cognitive impairment may forget to brush their teeth or forget that they have already done so, leading them to brush less or more frequently than cognitively healthy counterparts [22,23,24]. Meanwhile, attention deficits may impair their ability to remain focused on the goal of toothbrushing, resulting in premature termination of brushing, skipping steps or areas and reduced brushing quality. As cognitive impairment progresses, individuals may demonstrate more functional deficits. For instance, individuals with moderate cognitive impairment may lose track of the brushing process due to impaired working memory, leading to repetitive, fragmented, or incomplete brushing behaviors [25]. Increased attention deficits can lead to task interruptions or distractibility, making it difficult to maintain focus on the goal of cleaning all tooth surfaces. These further compromise the quality and thoroughness of toothbrushing. Individuals with severe cognitive impairment can struggle to initiate toothbrushing due to impaired executive functioning and impaired long-term motor memory [9,10,11]. As a result, these individuals are not only less likely to brush their teeth daily [26], but they also tend to lose independence in performing oral self-care [27] and often require various prompts to initiate and/or complete this task [9,10,11]. Apraxia (impaired praxis), which typically emerges in the middle to later stages of dementia, impairs the ability to carry out purposeful, coordinated movements despite having the desire and the physical ability to do so. This can result in difficulty initiating toothbrushing, halting the toothbrush in between movements, and completing the activity efficiently or in a timely manner [14,28,29]. All of these functional deficits can occur regardless of an individual’s physical or sensory capabilities and may be compensated for through environmental cues (e.g., a toothbrushing sequencing chart or leaving oral care supplies out in plain sight near the sink) and caregiver support.

As a result of impaired toothbrushing ability and altered daily routine, oral hygiene is often poor in older adults with cognitive impairment, especially those with moderate or severe cognitive impairment [27,30,31]. Increased plaque buildup elevates the risk of dental caries and periodontal infection in these individuals [32,33,34,35]. Older adults with cognitive impairment experience more caries (mean number of decayed coronal and root surfaces, 7.0 vs. 2.7) [36], periodontal disease (Relative Risk = 2.5, 95% CI 1.5–4.1) [35], loss of all natural teeth (63.3–72.7% vs. 42.6%) [35], and denture-related issues (24% vs. 7%) [37] than their healthy counterparts. Meanwhile, poor oral health can in turn accelerate cognitive decline [38,39,40,41] and increase agitation and other behavioral symptoms in people with dementia [42]. It can also lead to malnutrition, aspiration pneumonia, cardiovascular disease, or death [43,44,45,46,47,48,49].

Previous studies suggest that impaired oral self-care function (e.g., inability to brush teeth independently and/or effectively) plays a critical role in the decline of oral health in older adults with dementia. Oral self-care function mediates the association between cognitive impairment and oral health decline in older adults with cognitive impairment [19,22,50]. However, how oral self-care function varies across different levels of cognitive function remains unclear. This information is essential for individualizing oral hygiene intervention and providing functionally tailored support for older adults with cognitive impairment and their caregivers. To address this gap, we conducted a pilot study to preliminarily describe toothbrushing ability and functional deficits among older adults with varying levels of cognitive function.

## 2. Materials and Methods

### 2.1. Study Design and Hypothesis

A cross-sectional study design was employed to describe oral self-care function in older adults with different levels of cognitive function. Based on the conceptual framework, we hypothesized that toothbrushing ability, a key aspect of oral self-care, declines with increasing cognitive impairment. The study protocol was approved by the University of Iowa Institutional Review Board.

### 2.2. Subject Recruitment, Sample Size and Inclusion Criteria

A convenience sample of 81 participants with varying levels of cognitive function was recruited from local communities, assisted living facilities, and nursing homes in central and eastern Iowa, USA between August 2018 and January 2021. Recruitment was conducted through university-wide emails, hospital newsletters, physician referrals, dental record searches, recruitment flyers, and caregiver support groups. As this is the first study of its kind, no prior data were available to support a formal sample size estimation. Therefore, this sample size was determined pragmatically, taking into account available resources and staff support. Among the candidate participants, 13 were excluded for not meeting one or more of the following inclusion criteria: (1) age 50 years or older; (2) for participants with cognitive impairment, a documented diagnosis of mild cognitive impairment (MCI) or Alzheimer’s disease and related dementias established by a physician; or, for cognitively healthy participants, no known cognitive impairment and a perfect score (score = 5) on the Mini-Cog cognitive screening [51]; (3) ability to provide informed consent or to obtain consent through a legally authorized representative or power of attorney (POA); (4) presence of at least one natural tooth; and (5) ability to speak English. Two withdrew from the study and did not complete the data collection. The final study sample consisted of 14 cognitively healthy older adults recruited from local communities and 51 participants with documented cognitive impairment recruited from community settings, memory care clinics, assisted living facilities, and nursing homes.

### 2.3. Data Collection

#### 2.3.1. Cognitive and Functional Screening

Before informed consent and data collection, individuals meeting the above inclusion criteria first underwent a cognitive screening using the Mini-Cog to confirm their cognitive status. For those with documented cognitive impairment, a cutoff score of <4 [52] was used to increase sensitivity (i.e., individuals scoring <4 were considered eligible). Individuals without known cognitive impairment were required to achieve a perfect score (i.e., score = 5) to be included in the healthy comparison group, in order to improve the specificity of group classification and minimize inclusion of individuals with subtle or undetected cognitive impairment. Individuals with severe sensory deficits (e.g., deafness, blindness), disruptive behaviors or physical disabilities that prevent them from performing toothbrushing independently, and those who required antibiotic prophylaxis before dental appointments were also excluded from the study.

#### 2.3.2. Sociodemographics, Medical History and Medications

After informed consent, community-dwelling participants and their family caregivers completed an interview that queried participants’ age, gender, race and ethnicity, education, and living arrangement (e.g., living independently or with others). Health history (e.g., type of dementia, depression, anxiety and other comorbidities) and prescription medication use were also collected during the interview. For institutionalized participants, these data were collected through a review of facility records. Using the Anticholinergic Drug Scale, each prescribed medication was assigned a score based on its anticholinergic properties [53]. Scores were summed to estimate the total anticholinergic burden for each participant, with higher scores indicating greater burden. The total number of medications was also calculated for each participant.

#### 2.3.3. Assessment of Cognitive Function and Overall Dental-Related Function

Consented participants completed a cognitive assessment using the Standardized Mini-Mental State Examination (SMMSE) [53]. Based on their performance, participants were classified into five groups: healthy comparison (SMMSE = 25–30), mild cognitive impairment (MCI, SMMSE = 25–30), mild dementia (SMMSE = 20–24), moderate dementia (SMMSE = 13–19) and severe dementia (SMMSE = 0–12). SMMSE scores overlapped between the healthy comparison and MCI groups, consistent with the known limited sensitivity of the SMMSE for detecting mild cognitive impairment [54,55]. To reduce potential misclassification, Mini-Cog scores were additionally used to differentiate cognitively healthy participants (Mini-Cog = 5) from those with MCI (Mini-Cog < 5). Participants’ ability to perform oral health-related activities was also assessed using the Dental Activity Test (DAT) [56], a performance-based measure in which structured tasks are scored dichotomously (pass/fail) and summed to yield a total score. Based on the total score, participants were classified into different functional levels, with lower scores indicating greater impairment.

#### 2.3.4. Assessment of Oral Health

Prior to the assessment of toothbrushing ability, all participants underwent a brief oral examination conducted by a calibrated geriatric dentist in a research office, participant’s home, adult day center, or long-term care facility, using a headlamp, dental mirror, periodontal probe, and dental explorer. Standardized clinical criteria for caries diagnosis (e.g., the International Caries Detection and Assessment System, ICDAS) [57] could not be fully applied due to the field-based setting, limited participant cooperation, or non-standard positioning (e.g., wheelchair seating). For this reason, we used a validated protocol designed specifically for assessing the oral health status of older adults with special needs, including those with dementia, in non-dental environments (e.g., homes, nursing homes and medical clinics) [56,58,59]. Oral exams assessed oral hygiene (i.e., Debris Index score [60] and Gingival Index score [61]), number of remaining teeth and number of decayed/broken teeth. Decayed/broken teeth were combined because it was not always feasible to reliably distinguish between advanced carious lesions and severely broken-down teeth or retained roots under the aforementioned constraints. Therefore, a combined category was used to enhance consistency in assessment across participants.

#### 2.3.5. Assessment of Toothbrushing Ability

A previously designed and pilot-tested protocol was used to assess participants’ ability to perform toothbrushing. This protocol was developed based on the conceptual model and a widely used functional assessment tool for patients with Alzheimer’s disease [62].

After the oral exam, a trained research assistant placed a toothbrush, toothpaste, a paper cup, and a paper towel in front of the participant and asked them to brush their teeth as they normally would. During the assessment, verbal, visual, and tactile cues, alone or in combination, were provided when participants were unable to initiate or complete the task independently. These cues were used to evaluate how participants responded and whether they were able to complete the task with assistance. For example, if a participant failed to initiate toothbrushing, the research assistant repeated the instruction while handing them the toothbrush (verbal and visual cues). If the participant still did not begin brushing, the assistant provided a tactile cue by holding the participant’s hand, placing the toothbrush in their mouth, and guiding the motion to initiate brushing. If the participant continually brushed one area and failed to move the toothbrush to other areas, a verbal cue was given to encourage brushing of all tooth surfaces. Participants’ oral hygiene was assessed pre- and post-toothbrushing using the protocol described above to evaluate brushing effectiveness.

Participants’ toothbrushing performance was videotaped, reviewed, and coded. Based on the conceptual framework (Figure 1), performances were scored across four domains—task initiation, task completion, thoroughness, and quality of brushing—with the need for and response to cues incorporated into the scoring system. Task initiation was rated as initiating brushing without a cue (score = 4), with a cue (score = 2), or not initiating despite cues (score = 0). Task completion reflected whether all steps were carried out independently (score = 4), with a cue (score = 2), or not completed despite cues (score = 0). Thoroughness was based on whether all teeth and surfaces were brushed without a cue (score = 4), with a cue (score = 2), or whether some areas were missed (score = 0). Quality of brushing was assessed using the Debris Index (range 0–6), recorded before and after brushing, with ratings of 0 = poor, 1 = fair, 2 = good, and 3 = excellent [63]. The total brushing score reflected overall oral self-care ability, with higher scores indicating better function.

#### 2.3.6. Staff Training and Calibration

Before data collection, research staff received training in cognitive assessment (led by ND, a neuropsychologist), performance-based functional assessment and cueing strategies (led by LJ, an occupational therapist specializing in dementia care), and the data collection protocol (led by XC and JML). This training was followed by 3–5 supervised simulated assessments with real-time feedback until staff demonstrated mastery. Inter-rater (Kappa = 0.84) and test–retest (Kappa = 0.88) reliability were established. The oral examiner (JML, a geriatric dentist) was also trained and calibrated following the same protocol (Kappa > 0.9).

The coder (JML) received additional training (by LJ and XC) prior to coding and scoring participants’ performance, followed by supervised coding with real-time feedback for several participants until mastery was achieved.

### 2.4. Statistical Analysis

Descriptive statistics were first used to depict general characteristics of the participants. Performance scores were calculated for each participant. The total score was the sum of the numeric scores of each domain described above, reflecting the overall toothbrushing ability. The percent score (i.e., preserved ability) is the individual score divided by the total possible score for this task. This decimal score ranged from 0 to 1, corresponding to the proportion/percent of points out of total possible points earned across the toothbrushing tasks. We primarily used this score in our modeling and plotting. Bivariate analyses were performed to explore the factors associated with the five participant groups. Fisher’s exact and Chi-square tests were used for categorical variables, while Kruskal–Wallis (overall) and Wilcoxon rank-sum (pairwise) non-parametric tests were used. Pairwise *p*-values between SMMSE groups were corrected for multiple comparisons within the table via the Holm method.

We plotted preserved ability vs. SMMSE score, both with and without splines in the fitted line. Both the spline and no-spline plots showed overall oral self-care ability declined slowly with the advancement of cognitive impairment. While the no-spline plots matched our regression approach closely, the spline plot was presented here since it allowed more curvature and fit the data more closely.

The multivariable analyses consisted of two parts: oral self-care function versus SMMSE, and oral self-care function versus oral health measures. Two multivariable models were conducted to examine the association between cognitive impairment and toothbrushing performance. In the first model, Poisson regression was used to model toothbrushing performance as a rate of correct performance relative to the total possible score (using the total score as an offset). The model adjusted for age, sex, and total anticholinergic burden (ACB) of medications, with cognitive status (Standardized Mini-Mental State Examination groups) included as a categorical predictor. The cognitively healthy group served as the reference category. In the second model, linear regression was performed with SMMSE score treated as a continuous variable, using the same covariates. Both models demonstrated a consistent pattern of declining toothbrushing performance with worsening cognitive function, supporting the robustness of the findings across model specifications. To explore the association between self-care function and oral health outcomes, we used linear regression (for Debris Index, Gingival Index and number of remaining teeth) and negative binomial regression (for number of decayed/broken), adjusting for age, gender, ACB and oral self-care function. SMMSE was not adjusted for in this analysis since it was highly correlated with the overall functional score. We also ran another set of the multivariable analyses by replacing the covariate ACB with the presence of depression (Yes/No). The results of both models were very similar. Therefore, only the results of the first set of models are presented here.

All tests utilized a significance level of 0.05, and Holm correction was used to adjust for multiple comparisons. Statistical analysis was performed using the statistical software R version 3.6.1.

## 3. Results

### 3.1. Characteristics of the Study Participants

Participants averaged 76.5 years old (Table 1); most were female (61.5%) and had completed high school or higher education (98.5%). Participants with moderate or severe dementia were older than those in the healthy comparison group and those with MCI (*p* = 0.002) and were more likely to reside in a long-term care facility (*p* < 0.001). Depression or other mental health conditions were commonly seen in the participants with cognitive impairment, especially those with mild dementia. Compared to the healthy comparison group, participants with cognitive impairment were on average taking more medications and had a higher anticholinergic burden, indicating a higher risk of xerostomia. This was particularly true in those with moderate dementia. As expected, cognitive function (*p* < 0.001) and dental-related function (*p* < 0.001) were significantly different between groups and worsened in those with moderate or severe dementia. Participants with poorer cognitive function also had poorer oral hygiene (i.e., a higher debris index score, *p* < 0.001), more severe gingival inflammation (i.e., a higher gingival index score, *p* < 0.001) and more decayed/broken teeth (*p* = 0.02).

### 3.2. Toothbrushing Ability, Efficiency and Cognitive Function

**Figure 2:**
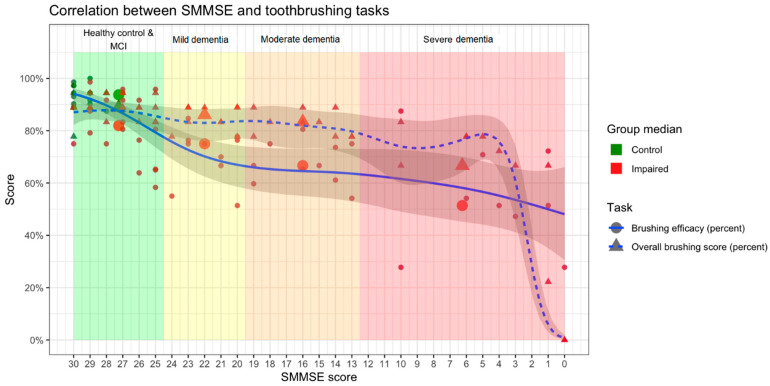
Toothbrushing ability and efficiency in participants with different levels of cognitive function. Note: SMMSE scores overlapped between the healthy comparison and MCI groups due to the limited sensitivity of the SMMSE for detecting mild cognitive impairment. The dashed and solid lines illustrate the trends in overall toothbrushing ability and toothbrushing efficacy, respectively, across different levels of cognitive function. Shaded areas represent 95% confidence intervals.

As shown in Figure 2, toothbrushing ability, as measured by the total brushing score, gradually declined alongside cognitive function until participants reached the profound stage (e.g., SMMSE ≤ 5), during which their toothbrushing ability dropped substantially and rapidly. After adjusting for age, sex, and anticholinergic burden, the MCI, mild dementia, moderate dementia, and severe dementia groups lost, on average, 3% (*p* = 0.8), 8% (*p* = 0.5), 12% (*p* = 0.3), and 37% (*p* < 0.001) of their toothbrushing ability, respectively, compared to the healthy comparison group (Table 2).

On the other hand, brushing efficiency, which was defined as 1 minus the sum of the plaque scores before and after brushing, divided by the total possible plaque score [6], declined early and rapidly in participants with cognitive impairment. Brushing efficiency declined by approximately 13% (*p* = 0.03) in the MCI group, 20% (*p* < 0.001) in the mild dementia group, 29% (*p* < 0.001) in the moderate dementia group, and 46% (*p* < 0.001) in the severe dementia group compared to the healthy comparison group (brushing efficiency score = 0.94).

### 3.3. Toothbrushing Quality, Cue Requirements and Cognitive Function

Table 3 shows that participants in the healthy comparison and MCI groups predominantly demonstrated excellent or good brushing quality, with all completing the task independently without any cues. The mild dementia group exhibited slightly reduced performance, with 80% rated as having good brushing quality and 20% as fair; however, all participants in this group also completed the task without assistance. In the moderate dementia group, brushing quality further declined, and a notable proportion required varying levels of assistance to complete the task. Most notably, 80% of participants in the severe dementia group required various cues to initiate and/or complete brushing, and 20% were unable to complete the task even with assistance. Brushing quality was significantly reduced in this group as well.

### 3.4. Main Functional Deficits Across Cognitive Levels

Main functional deficits and assistance needed to complete the brushing task are summarized in Table 4. Compared to the healthy comparison group, participants with MCI demonstrated reduced brushing time and efficiency, reflecting mild deficits in attention, executive function, and self-monitoring. In the mild dementia group, deficits were more pronounced than in the MCI group, with more frequent omission of surfaces, particularly lingual areas, suggesting early difficulties with sustained attention, working memory and visuospatial function. However, participants in both groups were able to complete the task independently.

In the moderate dementia group, participants exhibited greater deficits in attention, working memory, and executive function, resulting in both very brief and prolonged brushing, greater numbers of missed areas, and reduced overall efficiency. Loss of task sequence, random movement between quadrants, and other non-goal-directed brushing behaviors started to emerge in some participants. Although most participants were able to complete brushing independently, about 20% required cueing. Verbal (e.g., repeated instructions), visual (e.g., handing over the toothbrush), or tactile cues (e.g., hand-over-hand support) were often effective in helping participants initiate and re-engage with the task, after which they were generally able to proceed.

Participants with severe dementia presented with profound deficits across multiple cognitive domains, resulting in marked declines in toothbrushing ability. Due to severe executive dysfunction and impaired motor planning and sequencing function, task initiation was often absent or severely impaired. Fragmented, repetitive, and other non-goal-directed movements were frequently observed. When brushing occurred, it was usually brief, with many missed areas, leading to substantially reduced overall quality. At this stage, verbal or visual cues alone were often ineffective; combined verbal and visual or verbal and tactile cues were typically required to initiate toothbrushing. Even with such support, a considerable proportion of participants did not respond and had effectively lost the ability to brush their teeth independently.

### 3.5. Brushing Time and Cognitive Function

Brushing time tended to decrease with increasing cognitive impairment, except in the moderate dementia group, where a slight rebound in mean brushing time was observed (Table 5). On average, the estimated brushing time was 66.4 s in the healthy comparison group, 53.6 s in the MCI group, 31.9 s in the mild dementia group, 44.1 s in the moderate dementia group and 26.5 s in the severe dementia group (*p* = 0.10). The overall downward trend likely results from progressive declines in attention, working memory, and executive function, which lead to failure to maintain focus on the task goal, premature task termination, or incomplete brushing. The modest rebound observed in the moderate dementia group may result from disorganized or repetitive behaviors due to executive dysfunction and impaired working memory self-monitoring.

Further analysis showed that brushing time was significantly associated with overall toothbrushing ability (*p* = 0.02), brushing efficiency (*p* = 0.07), plaque score before brushing (*p* = 0.01) and gingival score (*p* = 0.07).

### 3.6. Toothbrushing Ability and Oral Health Measures

To examine the association between toothbrushing function and oral health measures, we categorized participants into high-, middle-, and low-function groups based on their total brushing scores. After adjusting for age, gender, and anticholinergic burden, participants with lower brushing ability had significantly poorer oral hygiene upon arrival. Specifically, the estimated debris index score before brushing increased progressively across groups: 0.34 (95% CI: 0.03–0.64) in the high-function group, 0.57 (95% CI: 0.42–0.73) in the middle group, and 1.22 (95% CI: 1.05–1.39) in the low-function group (*p* < 0.001). A similar trend was observed for estimated gingival index scores: 0.79 (95% CI: 0.43–1.15) in the high-function group, 1.03 (95% CI: 0.85–1.21) in the middle group, and 1.33 (95% CI: 1.13–1.53) in the low-function group (*p* = 0.02). On the other hand, the number of remaining teeth (*p* = 0.4) and the number of decayed or broken teeth (*p* = 0.9) did not differ significantly across groups.

## 4. Discussion

To our knowledge, this is the first study to detail toothbrushing ability and functional deficits among older adults across different levels of cognitive function. The findings revealed distinct patterns of oral self-care ability and functional impairment across cognitive levels. These results not only support our hypothesis and theoretical framework but also offer valuable insights to guide clinical interventions aimed at improving oral health in older adults with cognitive impairment.

First, this study revealed that the overall ability to perform toothbrushing tasks was relatively preserved through the earlier and moderate stages of impairment, but once individuals reached the profound stage, their oral self-care function deteriorated dramatically (Figure 2). This pattern is similar to the decline commonly observed in basic activities of daily living (ADLs), which also tend to decline slowly until the later stages of cognitive impairment [64]. However, when examining brushing efficiency, another key criterion for evaluating oral self-care function, a different pattern emerged. Impairments in brushing efficiency occurred early in the disease process and were more pronounced with the increasing severity of cognitive impairment. This pattern more closely resembles the trajectory of instrumental activities of daily living (IADLs), which usually begin to deteriorate in the earlier stages of cognitive impairment [64,65]. These findings suggest that beyond changes in toothbrushing patterns [22,23,24], diminished brushing quality can also serve as an early marker of functional deterioration in oral self-care. They further emphasize the need to distinguish between the ability to perform an oral self-care task, often overestimated by patients and caregivers, and the actual efficiency or quality of task performance, which is frequently overlooked [66,67]. Given the substantial local and systemic consequences of declining oral hygiene [68,69], brushing quality should be incorporated into clinical assessments of oral self-care function in older adults with cognitive impairment, rather than focusing solely on task performance.

The early decline in brushing quality also highlights the importance of establishing a caregiver–care recipient partnership in the early stage of the disease. This collaborative, reciprocal partnership is essential for maintaining oral health in older adults with cognitive impairment and would benefit patients as well as their family caregivers. First, it can help reestablish daily oral care routines for individuals whose oral self-care patterns are disrupted by memory loss, withdrawal, apathy/reduced motivation, or functional impairment that may occur in the early stages of disease [22,23,24]. This partnership also provides an opportunity for caregivers to monitor and ensure care quality for individuals with reduced brushing efficiency. For individuals with impaired functional independence, this partnership enables caregivers to provide essential support (e.g., prompting patients to initiate oral care, preparing oral hygiene supplies, or assisting with toothbrushing) on a regular basis, which is critical for preventing oral diseases and related systemic complications. Our study demonstrated that, when roles and expectations are clearly defined and mutually agreed upon, this partnership can also help care recipients maintain a sense of autonomy while alleviating caregivers’ concerns about overstepping boundaries, thereby enhancing caregiver–care recipient relationships (unpublished data).

In addition, the distinct functional deficits observed across cognitive levels suggest that the assistance needed to maintain oral hygiene varies among older adults at different stages of impairment. Accordingly, the level of caregiver support required may also differ across stages of cognitive impairment. Therefore, a personalized, family-centered approach is needed to provide functionally tailored oral care rehabilitation to help patients retain, regain, or improve self-care abilities, along with needs-based training and support to enhance caregiver efficiency. Specifically, for individuals with MCI or mild dementia, oral self-care functions are largely preserved. However, impaired sustained attention and working memory may result in reduced brushing quality. Therefore, oral health interventions (e.g., hands-on, interactive training on toothbrushing and interdental brushing) should primarily target patients, aiming to help them maintain or restore self-care function and improve care quality. At the same time, behavioral strategies and technology-assisted approaches to reestablish oral care routines (e.g., AI-based electronic reminder systems for dementia care [70] or pairing toothbrushing with a preferred daily activity) and strategies to monitor care quality (e.g., using plaque-disclosing solutions or recognizing signs of poor oral hygiene) can be provided to family caregivers. When tolerated, smart toothbrushes equipped with built-in sensors and artificial intelligence can be used to monitor brushing behavior and provide real-time, personalized feedback, thereby improving the quality of care [71]. Additionally, prompting techniques and strategies to enhance communication and cooperation can be provided as needed to better support caregivers [72,73,74].

In the moderate stage of cognitive impairment, most individuals can still perform oral self-care independently, though some require assistance to initiate the task due to executive dysfunction [75]. Attention and working memory deficits can lead to fragmented, repetitive or other non-goal-directed brushing behaviors in some individuals [75,76], resulting in increased missed areas and reduced effectiveness. As a result, toothbrushing can be brief or prolonged, yet brushing quality is often poor. These findings highlight the need for greater caregiver support for these individuals. Interventions should, therefore, shift focus from patients to family caregivers, with the aim of preserving patients’ remaining oral care abilities and enhancing caregivers’ efficiency in providing support. For patients, due to impaired learning ability, oral self-care training and rehabilitation should prioritize maintaining existing skills and ensuring care quality (e.g., toothbrushing training described above), rather than introducing new, unfamiliar tasks (e.g., flossing or interdental brushing for those with no prior experience). Caregiver training should equip families with practical strategies. Useful techniques include visual cues (e.g., a sequencing chart, handing the toothbrush to facilitate task initiation), verbal cues (e.g., instructing the individual to brush longer in specific areas), and tactile guidance (e.g., hand-over-hand assistance) [32,72,77]. Brushing together at bedtime is a good strategy to reinforce daily oral hygiene and provide needed support to improve care quality [63]. For individuals with behavioral symptoms, strategies to improve communication and cooperation should be introduced to caregivers [72,73,74]. Where appropriate, caregiver training can extend to include assistance with toothbrushing, interdental cleaning, and denture care.

Individuals with advanced cognitive impairment typically require substantial caregiver support or complete dependence due to severe functional decline (Figure 2; Table 2 and Table 3). In addition to the strategies discussed above, task-prompting techniques [32,72,77] and training caregivers to perform toothbrushing and other oral care for their care recipients are essential. As disease awareness and language abilities deteriorate, patients may lose the capacity to perceive, recognize, or communicate their dental needs [78,79,80]. Families should therefore be educated to recognize behavioral changes and other indicators of odontogenic pain or infection [80,81], enabling timely access to professional dental care.

While this study provides valuable insights into the functional deficits across different stages of cognitive impairment and informs clinical practice and oral health interventions for older adults and their caregivers, several limitations may affect the generalizability of the findings. First, one of the major limitations of this study is the relatively small and uneven sample size across cognitive subgroups, which was mainly due to recruitment challenges during the COVID-19 pandemic, limited funding and staff support and the lengthy cognitive, functional, and oral assessments required of study participants. The small sample size may have reduced statistical power and contributed to considerable variability in the estimation of functional ability among participants with differing cognitive levels. It may also affect the generalizability of the results to broader populations of older adults with cognitive impairment. Therefore, the findings should be considered preliminary and interpreted with caution. Second, this study included participants residing in different living conditions. Variations in living environments and available support (e.g., informal caregiver support for community-dwelling individuals versus professional care in long-term care settings) may influence oral self-care performance. These contextual factors, including caregiving routines and levels of assistance, could affect real-life toothbrushing performance and may not be fully captured in the study setting. Third, the cross-sectional design provides only a single time-point view and does not allow conclusions about causality or changes over time. Therefore, all findings should be interpreted as associations rather than evidence of progression in oral self-care function across cognitive decline. Large-scale longitudinal studies are needed to establish temporal and causal relationships between cognitive impairment and functional deficits in oral self-care among older adults with dementia. Additionally, the non-significant associations observed between oral self-care function and dental caries may be attributed to two factors. First, some participants were recruited during their dental treatment at the University of Iowa College of Dentistry and Clinics. Dental caries may have been restored prior to the baseline oral examination. This could partially explain the lack of observed association between oral self-care function and this outcome. Second, given the field-based setting, several constraints, including limited participant cooperation, non-standard positioning (e.g., wheelchair seating), and the absence of compressed air and radiographic imaging, prevented full application of standardized diagnostic criteria for dental caries (e.g., ICDAS). As a result, non-cavitated lesions (ICDAS codes 1–2) were not systematically assessed. This limitation may have led to an underestimation of dental caries prevalence among participants with cognitive impairment, especially those with behavioral challenges. Finally, longitudinal studies are needed to further examine the impact of impaired oral self-care function on oral health, systemic health, and quality of life in older adults with cognitive impairment.

In conclusion, oral self-care and care quality vary widely with cognitive function, underscoring the need for early care partnerships, tailored oral self-care rehabilitation, and targeted caregiver training to better support older adults with cognitive impairment and their families.

## Figures and Tables

**Table 1:** Characteristics of the study participants.

Characteristic	Overall (*n* = 65)	Healthy Comparison ** (*n* = 14)	MCI ** (*n* = 20)	Mild Dementia ** (*n* = 10)	Moderate Dementia ** (*n* = 10)	Severe Dementia ** (*n* = 11)	*p* Value
**Sociodemographics**							
Age (Mean, SD)	76.5 (12.1)	68.9 (10.1)	73.2 (10.8)	76.8 (14.8)	82.9 (6.6)	86.8 (9.5)	0.00
Female (%)	61.5	71.4	55.0	60.0	40.0	81.8	0.30
High school or higher (%)	98.5	100	100	90.0	100	100	0.40
Living condition							
Live alone at home (%)	39.1	100	50.0	10.0	0	0	<0.01
Live at home with others (%)	31.1	0	45.0	50.0	44.4	18.2	
Assisted living or nursing homes (%)	30.0	0	5.0	40.0	55.6	81.8	
**Medical history and medication**							
Depression (Yes, %)	22.3	14.3	10.5	50.0	33.3	22.2	0.14
Bipolar (Yes, %)	3.3	0	10.5	0	0	0	0.80
Number of mental health conditions (Mean, SD)	4.1 (4.0)	0	6.4 (4.5)	5.3 (3.4)	4.4 (3.4)	3.6 (2.8)	<0.01
Number of medications (Mean, SD)	5.4 (3.5)	2.6 (2.2)	5.4 (3.4)	7.1 (3.1)	8.0 (4.5)	5.6 (1.7)	0.00
Anticholinergic burden (Mean, SD)	1.4 (2.2)	0.4 (1.2)	1.5 (2.7)	1.7 (1.4)	2.9 (3.0)	0.9 (1.1)	0.04
**Cognitive and dental related function**							
SMMSE (Mean, SD) *	21.1 (9.2)	29.5 (0.7)	26.9 (1.6)	21.7 (1.5)	15.7 (2.3)	4.2 (3.6)	<0.01
Dental Activity Test score (Mean, SD)	6.9 (2.7)	8.9 (0.4)	8.2 (1.0)	7.8 (1.2)	6.1 (1.2)	1.8 (2.0)	<0.01
**Oral health measures**							
Debris Index score before brushing (Mean, SD)	0.9 (0.5)	0.3 (0.2)	0.7 (0.4)	1.1 (0.4)	1.1 (0.3)	1.5 (0.6)	<0.01
Gingival Index score (Mean, SD)	1.2 (0.5)	0.7 (0.3)	1.1 (0.4)	1.3 (0.2)	1.4 (0.3)	1.5 (0.6)	<0.01
Number of remaining teeth (Mean, SD)	19.8 (6.2)	22.7 (2.4)	18.3 (7.4)	19.5 (5.0)	18.6 (7.2)	20.1 (6.8)	0.15
Number of decayed/broken teeth (Mean, SD)	1.3 (2.7)	0.4 (1.6)	0.6 (0.8)	1.0 (2.0)	1.4 (1.5)	3.6 (5.2)	0.02
Use of removable dental prostheses (Yes, %)	15.0	0	29.4	12.5	20.0	9.1	0.20

Note: Total may not be 100% due to missing data. * SMMSE—Standardized Mini-Mental State Examination; ** Healthy comparison SMMSE = 25–30, mild cognitive impairment (MCI) SMMSE = 25–30, mild impairment SMMSE = 20–24, moderate impairment SMMSE = 13–19, severe impairment SMMSE = 0–12.

**Table 2:** Overall toothbrushing ability across cognitive levels estimated using Poisson regression.

Variable	Unadjusted IRR (SE, 95% CI)	*p*-Value	Adjusted IRR (SE, 95% CI)	*p*-Value
Healthy comparison	Ref	-	Ref	-
MCI	1.00 (0.09) (0.84 to 1.18)	1.0	0.97 (0.09) (0.81–1.17)	0.8
Mild dementia	0.96 (0.10) (0.78–1.17)	0.7	0.92 (0.12) (0.73–1.15)	0.5
Moderate dementia	0.93 (0.11) (0.76–1.14)	0.5	0.88 (0.13) (0.68–1.14)	0.3
Severe dementia	0.63 (0.12) (0.50–0.78)	<0.001	0.63 (0.14) (0.48–0.82)	<0.001
Age	-	-	1.00 (0.00) (1.00–1.01)	0.6
Gender: Male	-	-	1.07 (0.08) (0.92–1.24)	0.4
Anticholinergic burden score	-	-	1.01 (0.02) (0.97–1.04)	0.6

**Table 3:** Toothbrushing quality and cue requirements across cognitive groups.

Groups	Brushing Quality *	Completed the Task Without Any Cue	Completed the Task with Verbal Cue Only or Verbal and Visual Cues	Completed the Task with Tactile and Verbal Cues	Unable to Complete the Task with Cue(s)	*p* Value
Healthy comparison (*n* = 14)	Excellent	3 (21.4%)	0	0	0	<0.001
	Good	11 (78.6%)	0	0	0	
	Fair	0	0	0	0	
	Poor	0	0	0	0	
MCI (*n* = 20)	Excellent	4 (20%)	0	0	0	
	Good	16 (80%)	0	0	0	
	Fair	0	0	0	0	
	Poor	0	0	0	0	
Mild dementia (*n* = 10)	Excellent	0	0	0	0	
	Good	8 (80%)	0	0	0	
	Fair	2 (20%)	0	0	0	
	Poor	0	0	0	0	
Moderate dementia (*n* = 10)	Excellent	0	0	0	0	
	Good	5 (50%)	1 (10%)	1 (10%)	0	
	Fair	3 (30%)	0	0	0	
	Poor	0	0	0	0	
Severe dementia (*n* = 10) **	Excellent	0	0	0	0	
	Good	0	1 (10%)	1 (10%)	1 (10%)	
	Fair	2 (20%)	1 (10%)	2 (20%)	0	
	Poor	0	1 (10%)	0	1 (10%)	

* Quality of brushing was assessed using the Debris Index (range 0–6), recorded before and after brushing, with ratings of 0 = poor, 1 = fair, 2 = good, and 3 = excellent [62]. ** Excluded one participant who refused oral examination and did not perform brushing task.

**Table 4:** Main functional deficits and assistance requirements across cognitive levels.

	MCI	Mild Dementia	Moderate Dementia	Severe Dementia
Key functional deficits	Reduced brushing time Decreased brushing efficiency	Reduced brushing time Decreased brushing efficiency Missed tooth surfaces or quadrants	Brushing time was either brief or prolonged in some cases Increased missed areas Fragmented, non-goal-directed brushing behaviors in some participants Decreased brushing efficiency	Severely impaired or absent task initiation Brushing, if it occurred, was usually brief, with some prolonged episodes Fragmented, non-goal-directed brushing behaviors Increased missed areas Poor overall brushing quality
Assistance needed	Not observed	Not observed	Verbal, visual, or tactile cues might be needed to initiate brushing Participants typically responded well to cues and completed the task	Most required assistance to initiate toothbrushing Combined verbal + visual or verbal + tactile cues are often necessary Participants with profound impairment were unable to initiate brushing despite cues

**Table 5:** Brushing time and cognitive function.

Cognitive Level	Unadjusted Mean (SE)	95% Confidence Interval	Adjusted Mean (SE)	95% Confidence Interval	*p* Value
Healthy comparison (SMMSE = 25–30)	65.9 (9.7)	46.6–85.2	66.39 (10.85)	44.62–88.15	0.10
MCI (SMMSE = 25–30)	60.5 (8.1)	44.3–76.6	53.57 (8.62)	36.28–70.87	
Mild dementia (SMMSE = 20–24)	29.5 (11.4)	6.7–52.4	31.93 (11.43)	8.99–54.86	
Moderate dementia (SMMSE = 13–19)	42.1 (11.4)	19.3–64.9	44.08 (11.74)	20.53–67.63	
Severe dementia (SMMSE = 0–12)	27.8 (10.9)	6.0–49.6	26.50 (12.92)	0.58–52.42	

## Data Availability

The datasets presented in this article are not available because they are owned by the University of Iowa. Requests to access the datasets should be directed to Dr. Xi Chen.

## Abbreviations

The following abbreviations are used in this manuscript:

SMMSE	Standardized Mini-Mental State Examination
MCI	Mild cognitive impairment
ACB	Anticholinergic burden
DAT	Dental Activities Test
ICDAS	International Caries Detection and Assessment System
